# Oocyte ERM and EWI Proteins Are Involved in Mouse Fertilization

**DOI:** 10.3389/fcell.2022.863729

**Published:** 2022-03-14

**Authors:** J Cohen, L Wang, S Marques, C Ialy-Radio, S Barbaux, B Lefèvre, C Gourier, A Ziyyat

**Affiliations:** ^1^ Institut Cochin, INSERM, CNRS, Université de Paris, Paris, France; ^2^ Ecole Normale Supérieure (ENS), Université Paris Sciences et Lettres (PSL), CNRS, Sorbonne Université, Université de Paris, Paris, France; ^3^ Department of Histo-embryology, Genetics and Developmental Biology, Shanghai Jiao Tong University School of Medicine, Shanghai, China; ^4^ Service d’histologie, d’embryologie, Biologie de la Reproduction, AP-HP, Hôpital Cochin, Paris, France

**Keywords:** CD9, EWI-2, EWI-F, ERM, fertilization, microvilli, cortical tension

## Abstract

In mammalian fertilization, the link between the oocyte plasma membrane and underneath cytoskeleton has often been associated to key elements of successful gamete fusion, like microvilli shaping or CD9 function, but its effective role has poorly been studied. EWI-2 and EWI-F as cis partners of CD9, and ERM proteins (Ezrin, Radixin and Moesin) that both attach to the actin cytoskeleton and to the EWI are part of the molecules that make the link between the oocyte membrane and its cytoskeleton. This study aims to assay through siRNA inhibition, the involvement of these ERM and EWI molecules in mouse fertilization, their role in the microvilli morphology of the egg but also their possible contribution to the cortical tension, a parameter that reflects the mechanical behavior of the oocyte cortex. Whereas inhibiting separately the expression of each protein had no effect on fertilization, the combined inhibition of either EWI-2/EWI-F or the three ERM triggered a significant decrease of the fertilization index. This inhibition seems to correlate with an increase in the radius of curvature of the oocyte microvilli. It also causes a decrease of the oocyte cortical tension. These results show the importance of EWI-2 and EWI–F and ERM proteins in the smooth running of a fertilization event and support their involvement in the microvilli architecture of the oocyte and in its mechanical properties.

## Introduction

Fertilization is the process of union of two gametes. It occurs in several steps and culminates by the encounter of highly differentiated gametes. This interaction follows specific steps including recognition, adhesion and fusion. On the mammalian oocyte, JUNO and CD9 are the only two molecules known to be essential for adhesion/fusion during fertilization (Kaji et al., 2000; Le Naour et al., 2000; Miyado et al., 2000; Bianchi et al., 2014). While JUNO and its sperm partner IZUMO1 are now clearly identified as adhesion molecules (Aydin et al., 2016), the mechanism by which CD9 functions is still not completely understood, with most evidence pointing to an architectural or organizational role with other proteins embedded within the membrane (Runge et al., 2007; Jegou et al., 2011; Chalbi et al., 2014; Benammar et al., 2017). Indeed, the tetraspanin family, including CD9, contributes to the structural organization and plasticity of the plasma membrane. Regarding the architectural aspect, the concentration of CD9 molecules on microvillar membrane was found to be greater than that on the planar membrane. In addition, the complete absence of CD9 results in altered microvillar morphology, particularly a change of the radius of curvature of microvilli that are two fold higher than on wild-type oocytes (Runge et al., 2007) while a small radius of curvature is thought to promote fusibility (Wilson and Snell, 1998; McMahon and Gallop, 2005). An isolated large extracellular loop of CD9 was shown to inhibit fusion when preincubated with eggs, but not with sperm (Zhu et al., 2002) suggesting that CD9 is not a receptor for a sperm molecule. Only one article suggested that CD9 may function in a trans manner during gamete fusion by binding to an immunoglobulin (Ig) super family member, namely PSG17 (Ellerman et al., 2003). But this protein has never been found on sperm. Conversely, and in the sense of its cis action, CD9 associates with other tetraspanins, integrins, Igs and other adhesion receptors and proteins (reviewed in ((Charrin et al., 2014)). There are other members of Ig superfamily expressed by the oocytes with which CD9 interacts. Oocyte CD9 co-immunoprecipitates with two Ig superfamily cis partners, EWI-2 and EWI-F, which could have a role in linking CD9 to the oocyte microvillar actin core. Recently, crystal and cryo-electron microscopic structure of human CD9 and CD9-EWI-2 complex showed the interaction between CD9 and EWI-2, mediated by small residues in the transmembrane region and protein/lipid interaction suggesting the possible role of EWI-2 as a bridge between tetraspanins and other proteins, required for remodeling of membrane resulting in the formation of complex protein network. Cryo-electron microscopic structure of the CD9-EWI-2 complex and the mutation analysis revealed the importance of the small residues in the transmembrane region for the complex formation between CD9 and EWI-2 and the partial involvement of the CD9 large extracellular loop in the complex formation with EWI-2 (Umeda et al., 2020).

EWI-2 and EWI-F are tetraspanin CD9 and CD81 partners. They are immunoglobulins that share a Glu-Trp-Ile (EWI) extracellular motif. EWI-2 is also called IGSF8, PGRL, CD316 or KCT-4 and EWI-F is also known as FPRP or CD9-P1. Both associate directly with tetraspanins CD9 and CD81 while they do not associate with other tetraspanins or integrins (Charrin et al., 2001; Stipp et al., 2001; Sala-Valdes et al., 2006). In somatic cells, they induce tetraspanin relocalization into filopodia and they interfere in cell motility and aggregation (Charrin et al., 2001; Sala-Valdes et al., 2006; He et al., 2009). EWI-2 association with α-actinin regulates T cell immune synapses and HIV viral infection (Gordon-Alonso et al., 2012). Moreover, the expression level of membrane EWI-2 in *Cd9*-null oocytes is drastically reduced (He et al., 2009).

We have previously shown that CD9 generates fusion competent sites on the egg membrane for mammalian fertilization. These CD9-induced adhesion sites, named pro-fusional (i.e., able to lead to fusion), with strong adhesion, would be the actual location where fusion occurs. Indeed, in the absence of CD9, the adhesion between the two gametes exists but remains abortive (Jegou et al., 2011). Regarding the adhesive strength that we measured experimentally, the crucial difference between wild type (WT) and *Cd9*-null eggs comes from the strong interaction events that are absent when CD9 is absent. These adhesions are stronger because of the strong connection between the membrane and the cytoskeleton, that is not realized directly but through other molecules suggesting an involvement of CD9 in this process. Under these conditions, the link would likely occur via CD9-direct partners such as EWI-2 and EWI-F present in both somatic cells and oocyte (Rubinstein et al., 2006; Sala-Valdes et al., 2006; Runge et al., 2007). In turn, these CD9 partners bind directly to Ezrin-Radixin-Moesin (ERM) that connect the actin cytoskeleton to the plasma membrane (Sala-Valdes et al., 2006). Actin filaments cross-linked with the plasma membrane are important for generating cell-surface specialization areas. This is achieved through the regulation of actin-binding proteins. ERM proteins are thought to belong to the molecules linking the actin cytoskeleton and the plasma membrane. ERM proteins are widely distributed proteins located in the cellular cortex, in microvilli and adherens junctions. They contain a membrane binding domain and an actin binding domain. They have been implicated in mediating actin-membrane linkage and in regulating signaling molecules. In T lymphocytes for example, phosphorylated ERM are located at the posterior part of the cell where they have a potential role in the induction of uropod formation (Serrador et al., 1997; Lee et al., 2004). Indeed, ERM can have two conformations: a non-phosphorylated inactive cytoplasmic form and a phosphorylated active form located beneath the plasma membrane where it interacts with the cytoplasmic domain of adhesion proteins (For review (Louvet-Vallee, 2000; Fehon et al., 2010)). Furthermore, the function of lymphocyte Moesin is required for efficient HIV-1 viral fusion and infection (Barrero-Villar et al., 2009). More specifically, Dmoesin, the only member of the ERM family present in Drosophila, is required during oogenesis to anchor microfilaments to the oocyte cortex (Polesello et al., 2002) and *Ezrin*-deficient mice show microvilli disorganization in the developing intestine (Saotome et al., 2004). Mouse oocytes express all three ERM family members but seem to be particularly enriched in Radixin (Louvet et al., 1996; Larson et al., 2010). Finally, it has been shown that the disruption of ERM function reduces cortical tension in metaphase II eggs (Larson et al., 2010). It is known that cortical tension decreases during meiotic divisions and that a soft cortex is essential for asymmetric spindle positioning in mouse (Chaigne et al., 2013) which is guided by a narrow window of cortical tension (Chaigne et al., 2015). Recently it has been shown that aneuploidy in mouse oocytes can be generated by artificially decreasing cortical tension (Bennabi et al., 2020).

The purpose of our work was to analyze the role of ERM and EWI-2 and EWI-F proteins in gamete fertilization. We injected small interfering RNA (siRNA) to inhibit *ERM* and/or *EWI-2* and *EWI-F* gene expression into oocyte cytoplasm. To assess the functional and morphological effects of inhibition, we assessed the fertilization index and studied oocyte microvilli morphology and measured cortical tension.

## Materials and Methods

### Ethical Standards

All animal experiments were performed in accordance with national guidelines for the care and use of laboratory animals. Authorizations were obtained from local and governmental ethical review committees: Authorization APAFIS #14124-2017072510448522, A. Ziyyat (2018–2023).

### Sperm Preparation

Sperm from 8 to 10 weeks old male mice (B6CBAF1, Janvier, France) were expelled from cauda epididymis and vas deferens into a 500 μl drop of Ferticult medium (FertiPro N.V, Belgium) containing 3% BSA under mineral oil, resulting in a concentration of approximately 10^7^ sperm/ml. Sperm were then incubated at 37°C, 5% CO_2_ in air for 90 min to induce capacitation.

### Oocytes Retrieval and siRNA Micro-injection

**FIGURE 1:**
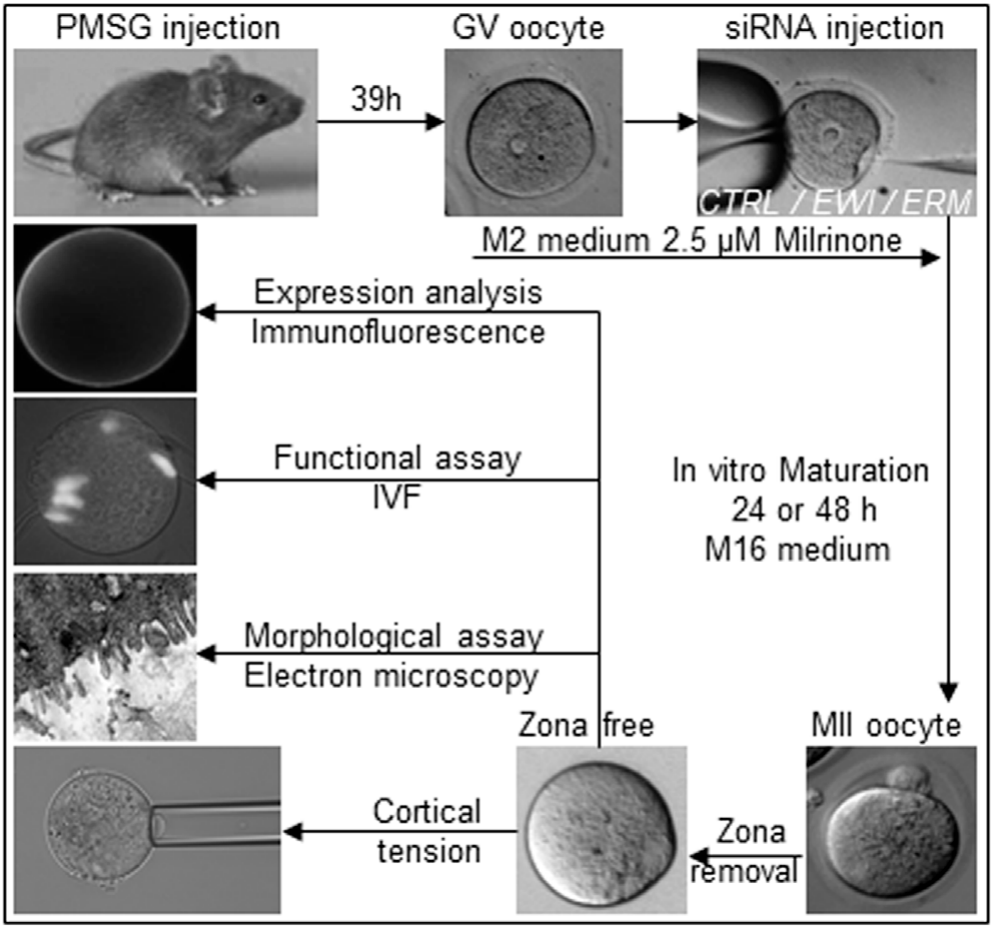
Experimental design. Germinal Vesicle stage (GV) oocytes were recovered from 5 to 8 weeks old B6CBAF1 mice 39 h post-PMSG injection. GV oocytes were maintained in M2 medium supplemented with 2.5 μM Milrinone to maintain meiotic arrest and placed on a Nikon inverted microscope at room temperature. Ten pl of siRNA solution (1 μM final concentration) were injected into the cytoplasm using a 1.2 µm inner diameter pipette connected to an injector. After microinjection, oocytes were washed several times to remove Milrinone and let in M16 medium to resume meiosis during 24 or 48 h at 37°, 5% CO_2_ in air. Then, after zona pellucida removing, oocytes were either assayed for their fertilizability or cortical tension measurement or fixed for electron microscopy or immunofluorescence studies.

Germinal Vesicle stage (GV) oocytes were collected from 5 to 8 weeks old B6CBAF1 (Janvier Labs, France) female mice at 39 h post-PMSG (Pregnant mare’s serum gonadotrophin, Intervet, France) injection. GV oocytes pick-up was performed in M2 medium (Sigma) supplemented with 2.5 μM Milrinone (Sigma) to maintain prophase I arrest. Cumulus cells were removed by repeated pipetting. Ten pl of siRNA solution were injected into the cytoplasm of denuded GV oocytes 1 hour after their retrieval, using a 1.2 µm inner diameter pipette (BioMedical Instruments, Germany) connected to a FemtoJet injector (Eppendorf, Germany) under a Nikon inverted microscope in the same M2 medium containing Milrinone at room temperature. Oocytes were then washed several times to remove Milrinone and matured *in vitro* in M16 medium (Sigma) during 24 or 48 h at 37°C, 5% CO_2_ in air. siRNA against *Ezrin* (sc-35350), *Radixin* (sc-36367), *Moesin* (sc-35956), *ERM* (sc-37850) *EWI-2* (sc-105564) and *EWI-F* (sc-142204) siRNA from Santa Cruz biotechnology were used at 1 μM final concentration. Control oocytes were injected following the same protocol with the same volume and concentration of control (*CTRL*) siRNA-A solution (sc-37007, Santa Cruz biotechnology). Figure 1 represents the experimental design of the study.

### Immunofluorescence Staining

Oocytes were fixed in PBS containing 1% BSA, 2% paraformaldehyde, 0.5% saponin (Sigma, France) (for membrane permeabilization when it was necessary). They were stained with anti-pERM antibodies at 5 μg/ml (#3726, Cell-Signaling technology), anti-Radixin at 4 μg/ml (Santa-Cruz biotechnologies), anti-EWI-2, anti-EWI-F at 5 or 10 μg/ml respectively (R&D systems, United States) or rat monoclonal anti-mouse CD9 at 20 μg/ml (KMC8; BD PharMingen, San Diego, MD, United States). Secondary antibodies used were anti-rabbit antibody for pERM at 4 μg/ml (GE Healthcare, United Kingdom), anti-goat Alexa 488 at 10 μg/ml for Radixin (Invitrogen, United States), anti-goat IgG TRITC Conjugate for EWI-2 at 2 μg/ml (ImmunoReagents, Inc, United States), anti-sheep Alexa 488 at 1 μg/ml for EWI-F (Molecular Probes, United States) and goat anti-rat Alexa green antibody for CD9 at 10 μg/ml (Molecular Probes). Stained eggs were then washed and mounted in Vectashield/DAPI for observation under UV light (Nikon Eclipse 600 microscope). Images of stained eggs were collected using the NIS-Elements D (Nikon) software and analyzed using ImageJ. Integrated density measurements of the entire oocyte were taken, using the elliptical selection tool to draw an ellipse around the cell. In order to eliminate any size-effect of the measured oocytes, we divided each result by the pixel size of the area analyzed. Results were first expressed in intensity per 10^5^ pixels then bring to a percentage considering the *CTRL* siRNA group at 100%.

### Zona Pellucida Removal and *in vitro* Fertilization

Oocytes were assessed after 24 h in M16 medium. Only Metaphase II (MII) oocytes were selected on the basis of the presence of the first polar body. Depending on the experiment, at 24 or 48 h, the zona pellucida (ZP) was subsequently removed by rapid treatment (<30 s) with acidic Tyrode’s solution (Sigma). Oocytes were then incubated for 2 h at 37°C, 5% CO_2_ in air to recover from the treatment, contemporaneously to sperm capacitation. ZP-free MII oocytes were washed and then placed in a 100 μl Ferticult medium drop containing 3% Bovine Serum Albumin (BSA, Sigma). ZP-free MII oocytes were inseminated with capacitated sperm for 2 h at a final concentration of 10^5^/ml, then washed and mounted in Vectashield/DAPI for observation under UV light (Nikon Eclipse 600 microscope). The fertilization index was evaluated as the total number of fused sperm/total number of eggs.

### Microvilli Morphology Assessment

#### Transmission Electron Microscopy

For transmission electron microscopy, *ERM*, *EWI* (*EWI-2* and *EWI-F*), *Mix* (*ERM* and *EWI*) or *CTRL* siRNA injected oocytes were fixed in a 100 µl drop of 0.25% glutaraldehyde (Sigma) in PBS 1% BSA for 30 min. After three washes in PBS 1% BSA, oocytes were fixed in 2.5% glutaraldehyde in Sorensen buffer supplemented with 1% BSA for 30 min at RT and 1 h at 4°C. After three washes in Sorensen buffer with 1% BSA, oocytes were post-fixed with 1% osmium tetroxide in 0.1 M phosphate buffer and then dehydrated in 70, 90 and 100% ethanol. After 10 min in a 1:2 mixture of epoxy propane and epoxy resin, oocytes were embedded in gelatin capsules with freshly prepared epoxy resin and polymerized at 60°C for 24 h. Samples were then mounted into epon blocks and sections of 90 nm were performed with an ultramicrotome (Reichert ultracut S), stained with uranyl acetate and Reynold’s lead citrate, and observed under a transmission electron microscope (Jeol 1011 microscope), with a Gatan camera. Pictures were taken all around the perimeter of each section.

### Measuring of the Microvillar Curvature

The radius of curvature at the tips of microvilli was determined by analyzing the images using the ImageJ software. The radius of curvature was determined to be the radius of a circle that fits the curve at the tip of the microvillus and correspond to the half of the diameter as illustrated in the zoomed area (Figure 6) as described previously (Runge et al., 2007). Only microvilli with distinct tips were used.

### Micropipette Aspiration for Cortical Tension Measurement

Micropipette aspiration (MPA) was adopted to measure the oocytes cortical tension as previously described (Larson et al., 2010; Evans and Robinson, 2018). Briefly, the zona pellucida of oocytes was removed using acid Tyrode’s solution. Oocytes were transferred into M2 medium with 3% BSA. The temperature of the medium was maintained at 37°C. A glass micropipette of a 12∼15 μm diameter was connected to a water reservoir of adjustable height. The micropipette and the tubing connections for the entire system were tested to confirm that no air bubbles were present. Zero aspiration pressure was set prior to each experiment by checking the absence of visible flow inside the pipette. Then, we measured the critical aspiration pressure (Δp) according to which the length of the protrusion pulled into the micropipette (Lp) is equal to the pipette radius (Rp). The cortical tension (Tc) was calculated using the Law of Laplace (Δp = 2Tc (1/Rp − 1/Rc)), where Rc is the radius of the oocyte. Oocytes during MPA were observed and images were captured using an inverted microscope equipped with a charge-coupled device (CCD) camera. Tc were measured on the microvillar regions of oocytes.

### Statistical Analysis

To compare the fluorescence intensities, an unpaired Student t test was used. To compare the Fertilization Indexes (mean ± s. e.m.), a Mann-Whitney test was used. To compare the cortical tensions and microvilli radius, a one-way ANOVA test with multiple comparisons was used. Differences were considered significant at *p* < 0.05. All experiments were realized at least three times.

## Results

Figure 1 illustrates the experimental set-up including the different steps, ranging from oocytes preparation, siRNA injection, *in vitro* maturation and zona removal to the various tests that have been applied, namely immunofluorescence staining, zona-free *in vitro* fertilization assay, electron microscopy analyzes and measurement of cortical tension.

### *ERM* and *EWI-2* and *EWI-F* siRNAs Are Effective to Reduce Expression of Their Respective Proteins at the Oocyte Membrane Level

To evaluate the efficiency of siRNA inhibition on protein expression, we compared by immunofluorescence the microinjected GV oocytes, by one or a combination of siRNAs and then matured *in vitro*.

#### Effect of the *EWI-2* and *EWI-F* siRNA Injection on the Corresponding Protein Membrane Expression

**FIGURE 2:**
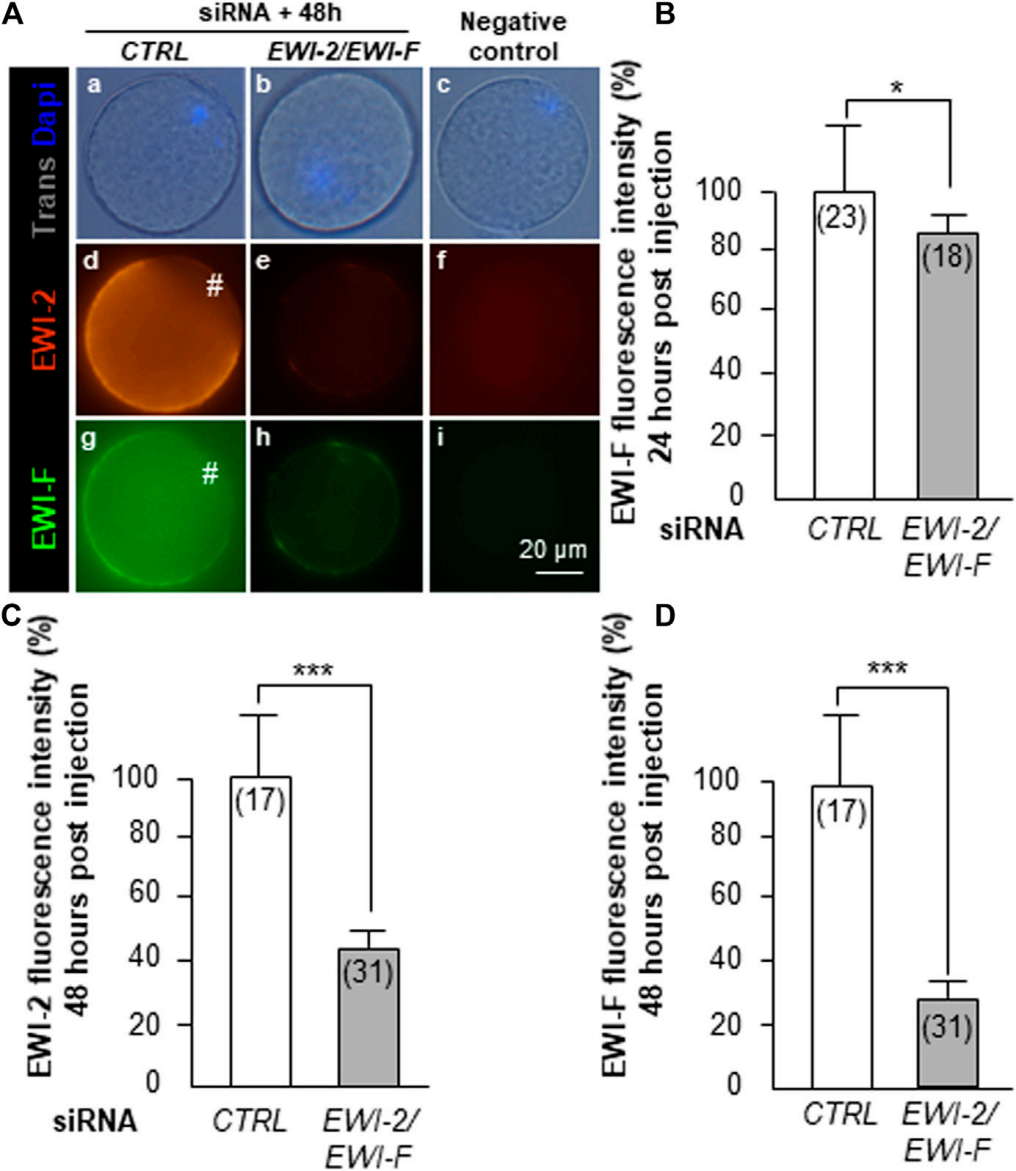
EWI-2 and EWI-F oocyte membrane expression 24 and 48 h post *EWI-2/EWI-F* siRNA injection. *CTRL* siRNA (A a,d,g) or *EWI-2/EWI-F* siRNA (A b,e,h) together were microinjected into GV oocytes, which were let to mature 24 or 48 h *in vitro*, to improve the expression inhibition. They were then fixed and incubated with the anti-EWI-2 or the anti-EWI-F antibodies and stained by the secondary antibodies anti-goat IgG TRITC or anti-sheep Alexa 488 respectively. Negative controls **(A)** c,f,i without primary antibodies. Analysis of the fluorescence intensity **(B,C,D)** was performed and *EWI-2/EWI-F* siRNA microinjected oocytes showed, after 24 h *in vitro* maturation, a weak (13%) but a significant (*p* = 0.02) decrease of fluorescence intensity of the EWI-F protein compared to *CTRL* siRNA group **(B)**. After 48 h *in vitro* maturation, greater reductions of the EWI-2 55%, **(C)** and EWI-F 68%, **(D)** staining compared to the *CTRL* siRNA injected oocytes were observed (*p* < 0.0001). # amicrovillar zona; ( ) number of oocytes.

When observed by epifluorescence after immunostaining 24 h after microinjection of combined *EWI-2* and *EWI-F* siRNA or *CTRL* siRNA, EWI-2 was localized on the egg membrane with no difference detectable to the naked eye between the two groups. However, further quantification of the fluorescence with ImageJ revealed a weak (13%) but significant (*p* = 0.02) decrease of fluorescence intensity of the EWI-F protein after *EWI-2/EWI-F* siRNA (n = 18) compared to *CTRL* siRNA (n = 23) injection (Figure 2B). This small reduction of EWI-F expression should be due to a poor efficiency of siRNA, to its degradation or to a high stability of the EWI-F protein.

To check this last point and try to improve the expression inhibition, we left the siRNAs acting for 48 h (instead of 24 h) before performing immunostaining of EWI-2 and EWI-F. After this delay, a clear reduction of expression of both EWI-2 and EWI-F was observed on the oocytes injected with *EWI-2* and *EWI-F* siRNA compared to the *CTRL* group (Figure 2A d vs. e and g vs. h). While negative controls (without primary antibodies) did not reveal any signal (Figure 2A f, i), significant decreases (55 and 68%; *p* < 0.0001; Figures 2C,D) in the fluorescence intensity of EWI-2 and EWI-F were observed respectively (n = 31) compared with *CTRL* siRNA oocytes (n = 17). This result suggested the stability of EWI-2 and EWI-F proteins.

#### *ERM* siRNA Injection Reduces Radixin and Phosphorylated ERM Membrane Expression

**FIGURE 3:**
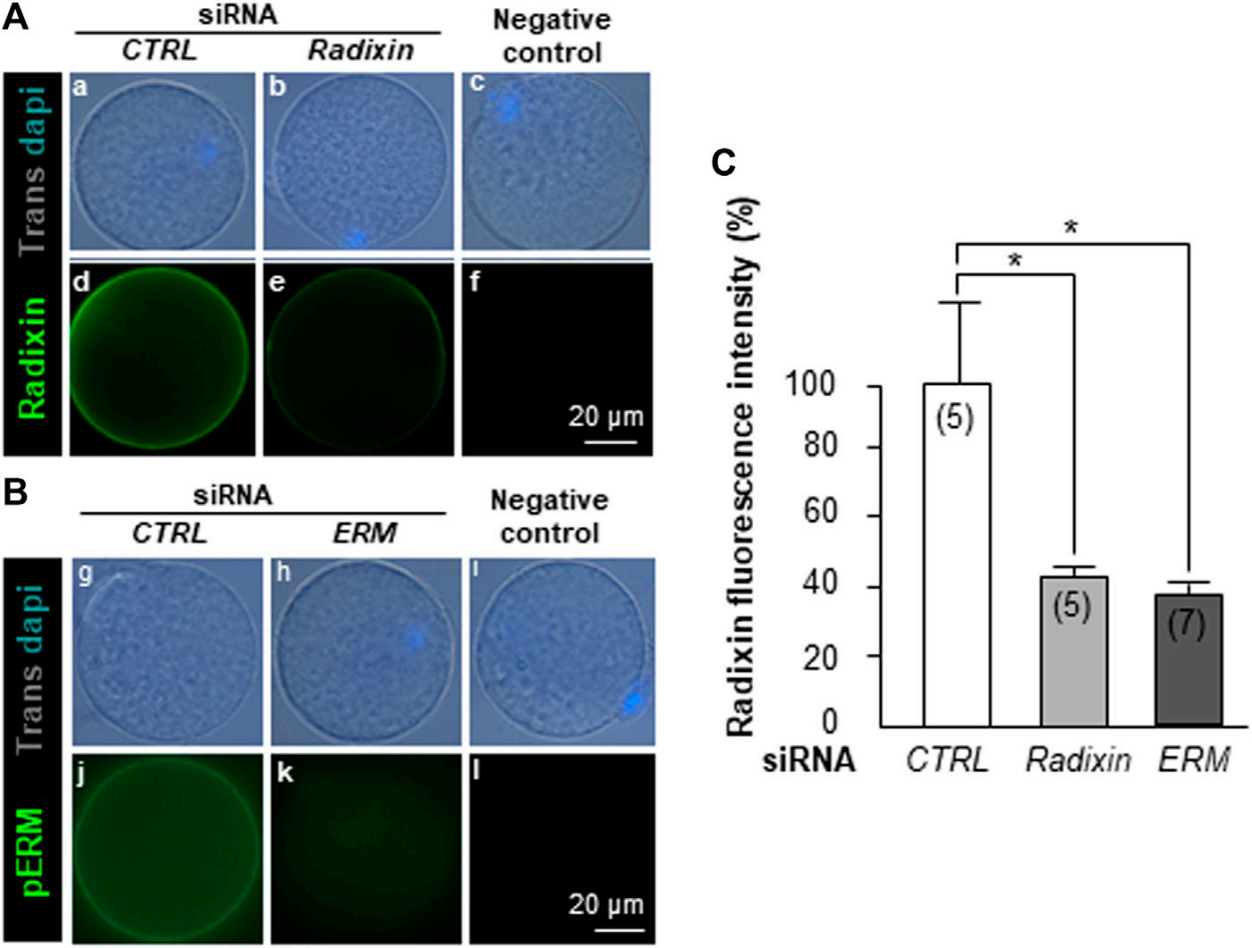
*Radixin* and *ERM* siRNA injection on Radixin and phosphorylated ERM (pERM) membrane expression, respectively. **(A)**. *CTRL* siRNA (a,d) or *Radixin* siRNA (b,e) were microinjected into GV oocytes, which were let to mature 24 h *in vitro*, then fixed, incubated with anti-Radixin antibodies and then with secondary antibodies anti-goat Alexa 488. Negative control without primary antibodies (c,f). **(B)**. *CTRL* siRNA (g,j) or *ERM* siRNA (h,k) were microinjected into GV oocytes, which were let to mature 24 h *in vitro*, then fixed, incubated with anti-pERM antibodies and then with secondary antibodies anti-rabbit Alexa 488. Negative control without primary antibody (i,l). **(C)**. *Radixin* siRNA compared to the *CTRL* siRNA injection resulted in a significant decrease (*p* = 0.03) of fluorescence intensity of Radixin. Combined *ERM* siRNA, compared to *CTRL* siRNA injection, dramatically decreased Radixin oocyte membrane expression. ( ) number of oocytes.

Three groups of GV oocytes were injected either with *Radixin*, or *Ezrin*, or *Moesin* siRNA, and 24 h later, they were immunostained with respective antibodies. The *Radixin* siRNA group (n = 5), showed a significant decrease (56%; *p* = 0.03; Figure 3C) of the fluorescence due to Radixin compared to the *CTRL* group (n = 5) (Figure 3A d vs. e). Unfortunately, because of nonspecific signals obtained with the antibodies directed against Ezrin and Moesin, we could not estimate the influence of *Ezrin* and *Moesin* siRNA on Ezrin and Moesin protein expression. To circumvent this difficulty, we injected combined *ERM* siRNA and 24 h later we used antibodies recognizing the phosphorylated form of the three ERM proteins (pERM). Immunostaining results showed a dramatic decrease of pERM membrane expression since no fluorescence could be detected in the *ERM* siRNA injected group (n = 13) contrary to the *CTRL* group (n = 20) (Figure 3B j vs. k). We also assessed the specific expression of Radixin at the oocyte membrane after *ERM* siRNA injection (n = 7). We found a significant decrease (62%) of its immunofluorescence intensity compared to the *CTRL* siRNA injected group (*p* = 0.01; Figure 3C).

### Combined, But Not Isolated *EWI-2* and *EWI-F* or *Ezrin*, *Radixin*, *Moesin* siRNAs Injection Decrease the Fertilization Ability of the Oocyte Without Decreasing CD9 Expression

#### Oocyte Fertilization After *EWI-2* and/or *EWI-F* siRNA Injection

**FIGURE 4:**
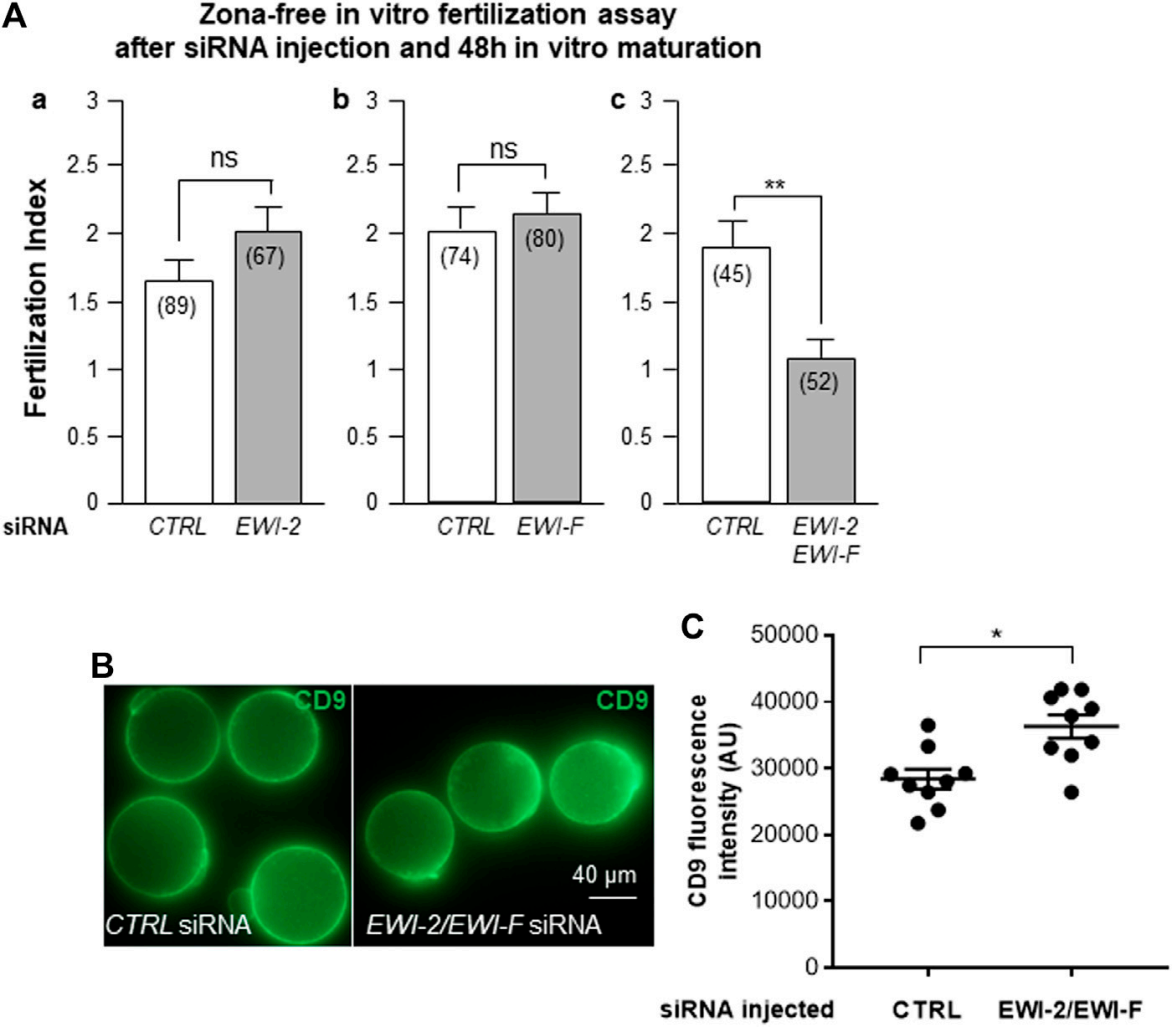
Combined, but not isolated *EWI-2* and *EWI-F* siRNAs injection on oocyte inhibits fertilization without disrupting CD9 expression. **(A)**. a. After *EWI-2* siRNA injection, *in vitro* maturation (48 h), zona removal and *in vitro* fertilization, the oocyte fertilization index observed was not different from the one of the *CTRL* siRNA oocyte group. b. After *EWI-F* siRNA injection, *in vitro* maturation (48 h), zona removal and *in vitro* fertilization, the oocyte fertilization index observed was not different from the one of the *CTRL* siRNA injected oocytes. c. After combined *EWI-2* and *EWI-F* siRNA injection, *in vitro* maturation (48 h), zona removal and *in vitro* fertilization, a significant reduction of the FI (43%, *p* = 0.0011) was observed compared to the *CTRL* siRNA group. ( ) number of oocytes from at least three different experiments. **(B)**. Images illustrating CD9 immunofluorescence on oocytes microinjected with *CTRL* or *EWI-2/EWI-F* siRNA and *in vitro* matured during 48 h. **(C)**. Statistical comparison (T-test) using ImageJ quantification of CD9 fluorescence intensity between *CTRL* and *EWI-2/EWI-F* siRNA groups (*p = 0.03).

After 48 h of *in vitro* maturation following *EWI-2* or *CTRL* siRNA injection, zona removal and *in vitro* fertilization, the fertilization indexes (FI) observed were 2.03 ± 0.19 (n = 67) and 1.66 ± 0.16 (n = 89) respectively (Figure 4Aa). After *EWI-F* siRNA injection, FI was 2.16 ± 0.15 (n = 80) compared to 2.03 ± 0.16 after *CTRL* siRNA injection (n = 74) (Figure 4Ab). These differences after *EWI-2* or *EWI-F* siRNA isolated injection were not significant.

On the contrary, simultaneous decrease in proteins EWI-2 and EWI-F expression after combined siRNA injection was associated with a significant reduction of the FI (43%, *p* = 0.0011). Indeed, fertilization index was 1.91 ± 0.19 sperm per egg in *CTRL* siRNA microinjected oocytes (n = 45) compared to 1.08 ± 0.14 in *EWI-2* and *EWI-F* combined siRNAs injected oocytes (n = 52) (Figure 4Ac).

#### Inhibition of EWI-2 and EWI-F did Not Decrease CD9 Expression

Considering that EWI-2 and EWI-F are direct partners of CD9, we sought to find out if their effects on fertilization were dependant on a reduction in the expression of CD9, itself directly involved in this process. Immunofluorescence of CD9 in oocytes microinjected with *CTRL* or *EWI-2/EWI-F* siRNA (Figure 4B) unexpectedly revealed a slight increase in CD9 expression in oocytes microinjected with *EWI-2/EWI-F* siRNA (Figure 4C). No difference in CD9 localization was observed between the two groups.

#### Combined Injection of *Ezrin, Radixin and Moesin* siRNA Decreases Fertilization Index

**FIGURE 5:**
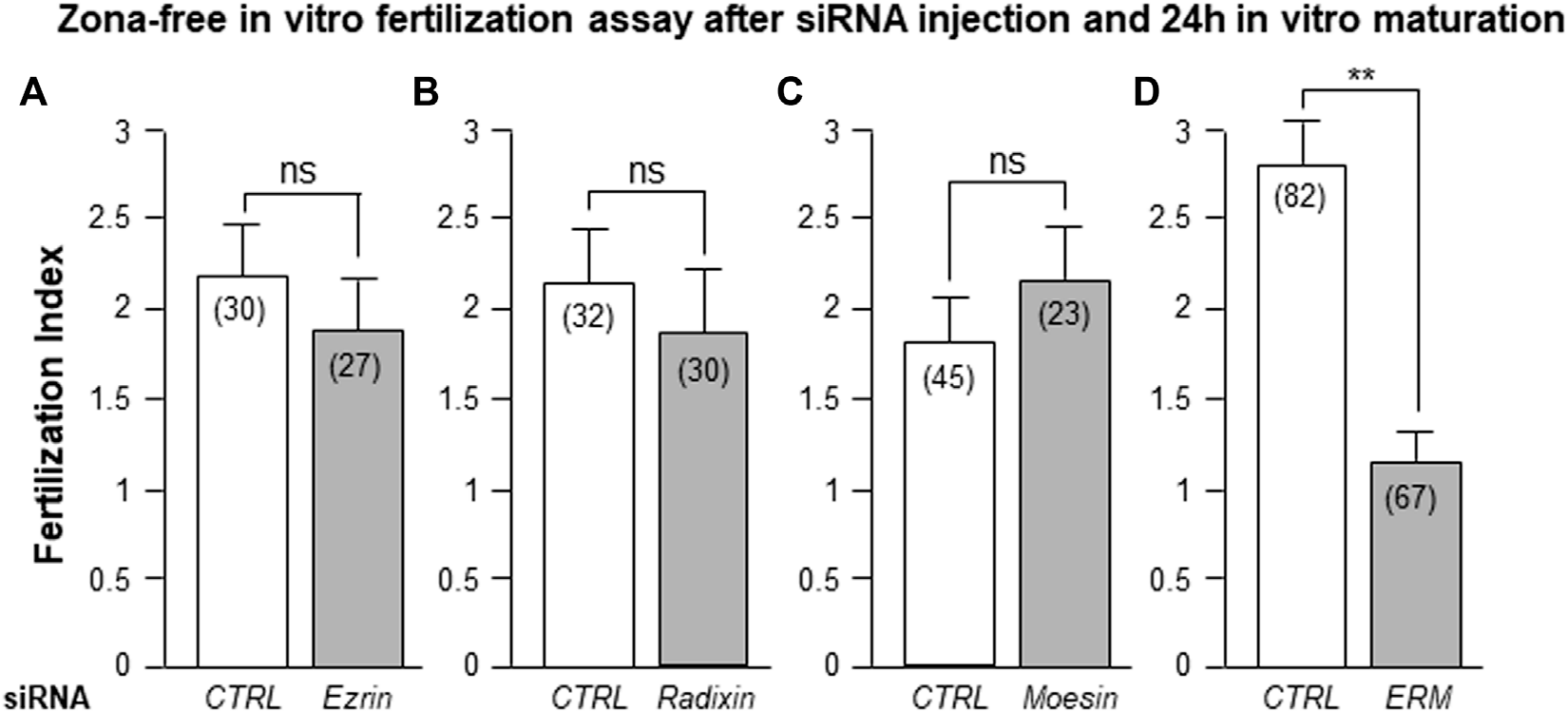
Combined, but not isolated, *ERM* siRNAs injection in oocyte inhibited fertilization **(A)**. After *Ezrin* siRNA injection, *in vitro* maturation (24 h), zona removal and *in vitro* fertilization, the oocyte fertilization index observed was not different from the one of the *CTRL* siRNA oocyte group. **(B)**. After *Radixin* siRNA injection, zona removal and *in vitro* fertilization, the oocyte fertilization index observed was not different from the one of the *CTRL* siRNA oocyte group **(C)**. After *Moesin* siRNA injection, zona removal and *in vitro* fertilization, the oocyte fertilization index observed was not different from the one of the *CTRL* siRNA oocyte group **(D)**. After combined *ERM* siRNA injection, zona removal and *in vitro* fertilization, a significant reduction of the FI (59%; *p* < 0.001) was observed compared to the *CTRL* siRNA group. ( ) number of oocytes from at least three different experiments.

As detailed in Figure 5, after 24 h of *in vitro* maturation, while isolated injection of either *Ezrin*, *Radixin* or *Moesin* siRNA did not affect the FI (Figures 5A–C), combined *ERM* siRNA injection did induce a significant decrease of the FI (59%) from 2.82 ± 0.23 after *CTRL* siRNA injection (n = 82) to 1.15 ± 0.16 (n = 67) (*p* < 0.001, Figure 5D).

### *EWI-2*, *EWI-F*, *ERM* or Combined (*EWI/ERM*) siRNAs Injection on Oocyte Alter Microvilli Morphology and Density

**FIGURE 6:**
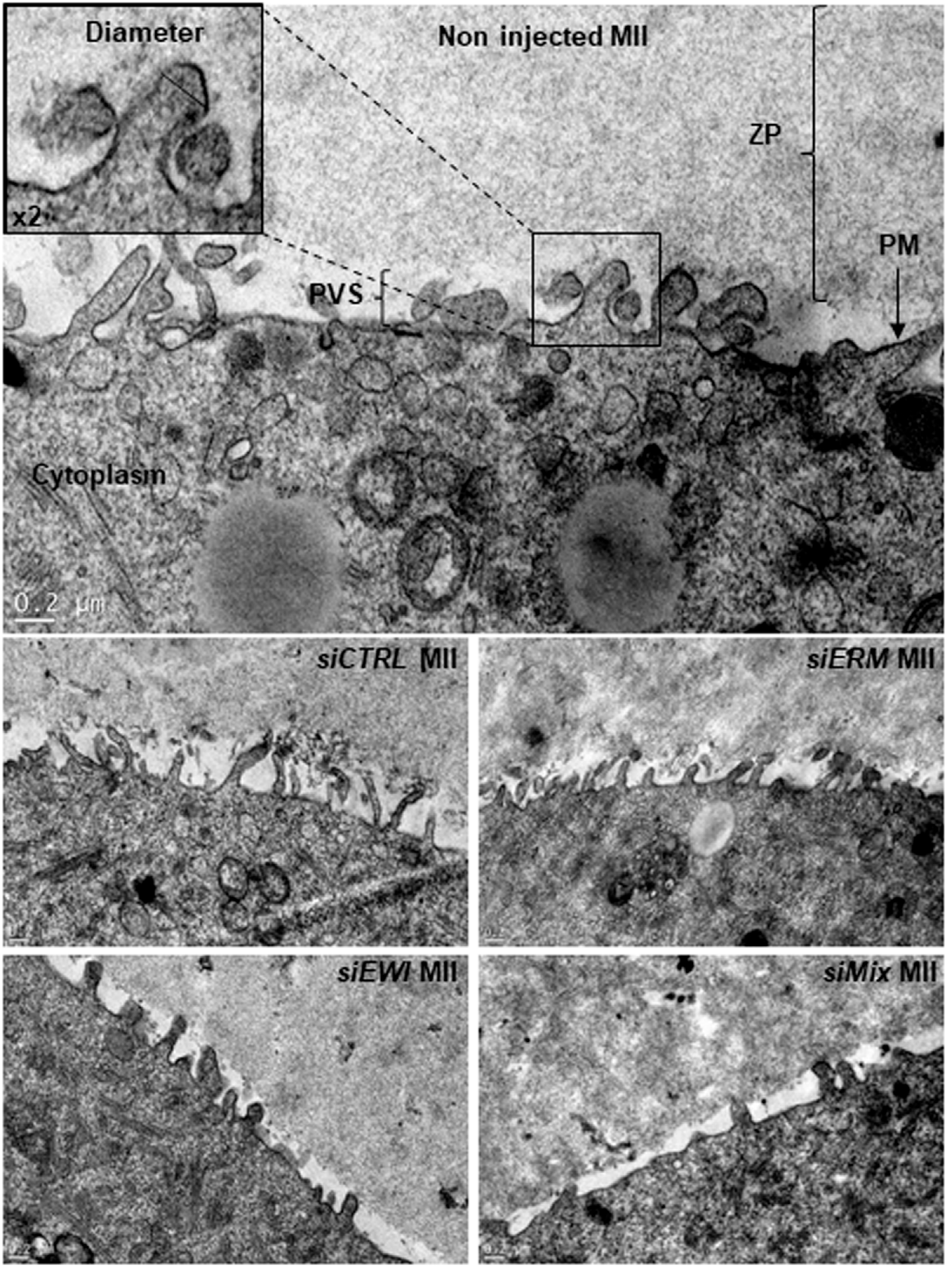
*ERM* and/or *EWI* siRNAs injection alters microvilli morphology. After *CTRL*, *ERM*, *EWI* or *Mix* siRNA injection and 48 h *in vitro* maturation, Metaphase II oocytes were fixed and their microvilli analyzed by electron microscopy. Zona Pellucida (ZP), Plasma Membrane (PM), Perivitelline Space (PVS), Cytoplasm and Radius of curvature are shown in a non-microinjected MII oocyte. An example image is shown for each microinjected MII oocyte group. Scale bar: 0.2 µm.

**FIGURE 7:**
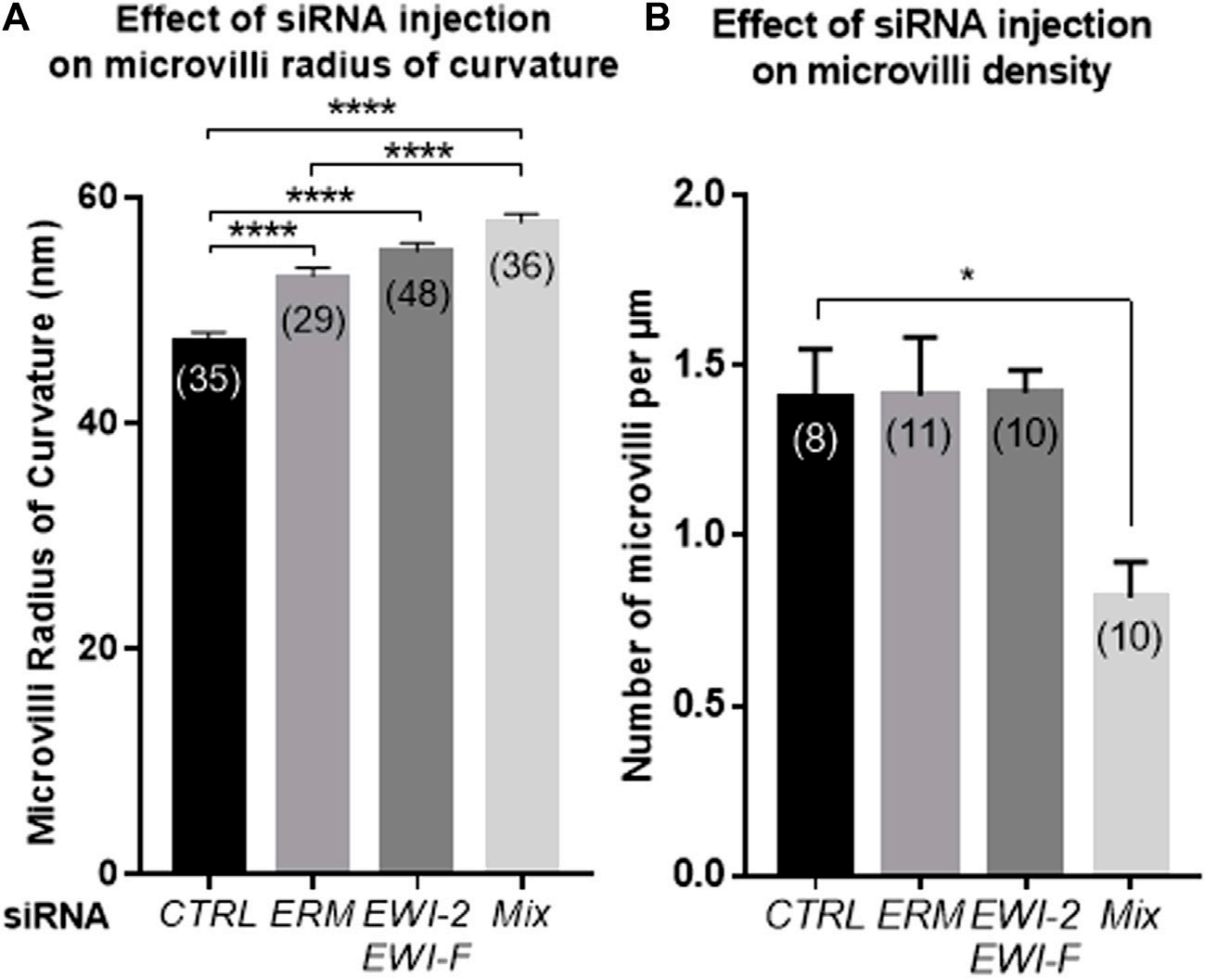
Effect of *CTRL*, *ERM*, *EWI* or combined *ERM-EWI* siRNAs injection on microvilli morphology. After siRNA injection and 48 h *in vitro* maturation, Metaphase II oocytes were fixed and their microvilli analyzed by electron microscopy. **(A)**. Microvilli radius of curvature increased after *ERM*, *EWI* or combined *ERM-EWI* (*Mix*) siRNAs injection and maturation compared to *CTRL* siRNA injected oocytes (****p < 0.0001). **(B)**. Decrease of microvilli density after *Mix* siRNA (combined *ERM* and *EWI* siRNA) injection and maturation (*p < 0.01). ( ): Oocyte number from at least three different experiments.

After *CTRL*, *ERM*, *EWI-2/EWI-F* or *Mix* (*ERM* and *EWI-2/EWI-F*) siRNA injection and 48 h *in vitro* maturation, we used transmission electron microscopy to analyze Metaphase II oocytes microvilli in terms of radius of curvature and density. Figure 6 provides a typical image of each group. The mean radius of curvature of the microvilli increased from 47.44 ± 0.63 nm for *CTRL* siRNA injected oocytes (553 microvilli on 35 oocytes) to 53.08 ± 0.75 nm (931 microvilli on 29 oocytes, *p* < 0.0001), 55.26 ± 0.69 nm (425 microvilli on 48 oocytes, *p* < 0.0001) and 57.78 ± 0.81 nm (493 microvilli on 36 oocytes, *p* < 0.0001) for *ERM*, *EWI-2/EWI-F* and *Mix* siRNA injected oocytes respectively. ANOVA tests show a significant increase of microvilli radius in all groups as compared to the *CTRL* siRNA group and between the *EWI-2/EWI-F* siRNA and *Mix* siRNA groups and between the *ERM* siRNA and *EWI-2/EWI-F* siRNA groups (Figure 7A). To estimate the actual error made during the measurements, we took at random two microvilli in each group that we measured three times. The average standard deviation between the three measurements ranged between 0.255 and 1.326 nm which is significantly lower than the reported difference between the groups.

Regarding the density of the microvilli, the only significant difference (*p* < 0.01) obtained was between the *Mix* siRNA group (0.81 ± 0.10/µm) and the three other groups (1.41 ± 0.13/µm, 1.41 ± 0.17/µm and 1.42 ± 0.06/µm for *CTRL*, *ERM* and *EWI-2/EWI-F* siRNA microinjected groups respectively). In each group, between 8 and 11 oocytes from at least three different experiments were analyzed. The counts were carried out on approximately 300 images per group that represent more than one thousand microvilli per group (Figure 7B).

### Partial Inhibition of ERM and EWI-2/EWI-F Expression Decreases Metaphase II Cortical Tension

Previous studies identified important changes in the cortical tension of oocytes during their progression through meiotic maturation and egg activation (Larson et al., 2010). It has also been shown that a perturbation of cortical tension through the function disruption of the family of actin-to-membrane tethering (ERM) causes significant defects in spindle function during exit from metaphase II arrest. Here we studied the effect of the inhibition of ERM, EWI-2/EWI-F expression on the oocyte cortical tension. When microinjected, the oocytes were at the germinal vesicle (GV) stage. Then, they were matured *in vitro* up to the MII stage. During the microinjection, few oocytes did not tolerate this mechanical trauma and lysed quickly. Those that resisted to this step survived and over 80% of them, regardless of the siRNA injected, matured and reached the MII stage. All microinjected oocytes were measured at MII stage.

**FIGURE 8:**
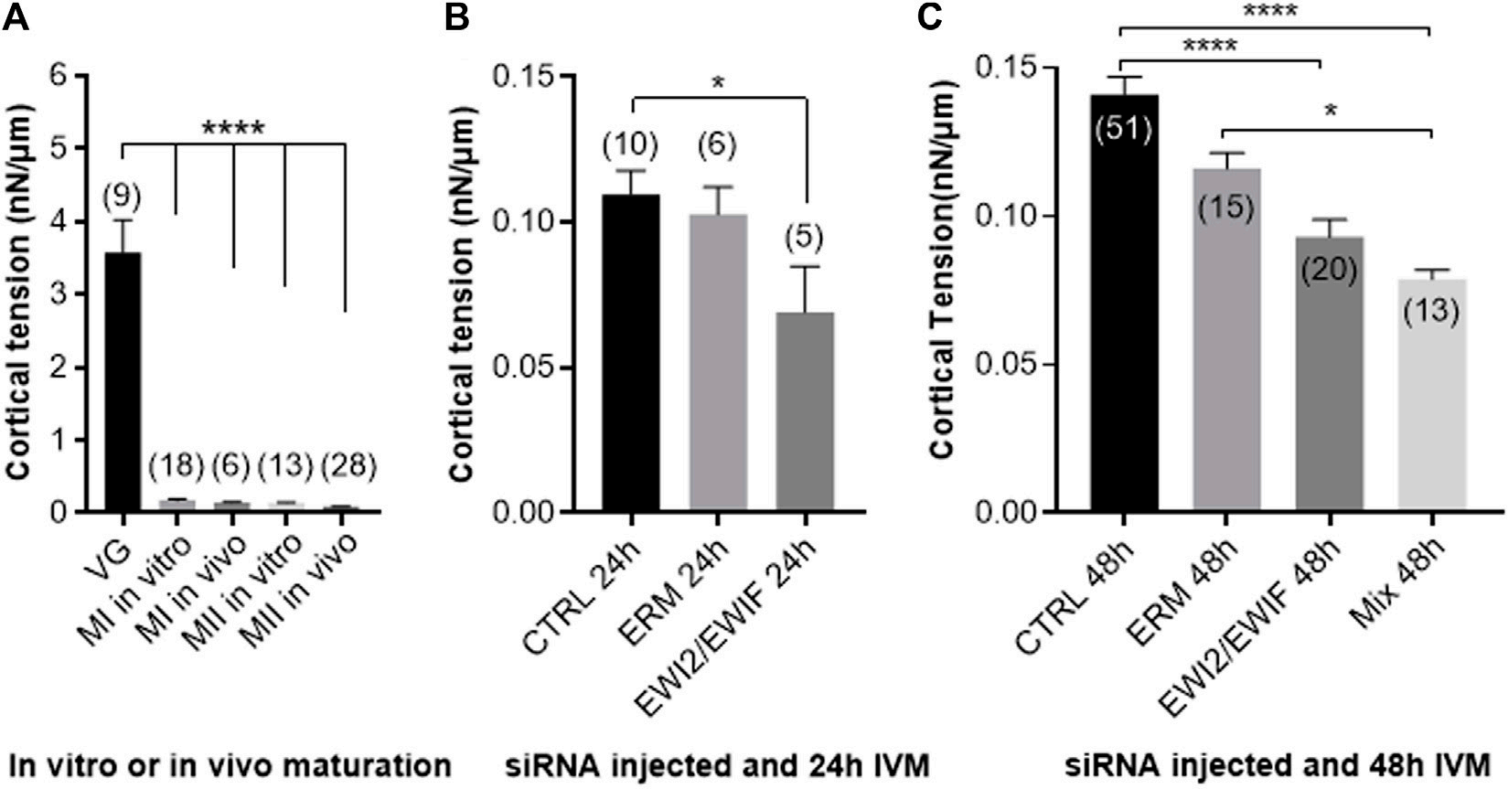
*ERM* and *EWI* siRNAs injection decreased oocyte cortical tension. **(A)** A drastic drop in cortical tension was observed between the Germinal Vesicle stage (GV) oocytes and metaphase I (MI) or metaphase II (MII) oocytes whether the latter were matured *in vivo* or *in vitro* without any injection (****p < 0.0001). **(B)**. A slight decrease in cortical tension was observed after injection of siRNA *EWI* (*EWI-2* and *EWI-F*) and 24 h of maturation compared to oocytes injected with *CTRL* siRNA (*p = 0.02). **(C)**. When the injected oocytes had matured for 48 h, the decrease of the cortical tension after the injection of siRNA *EWI* or siRNA Mix was more pronounced compared to the *CTRL* siRNA group (****p < 0.0001 and *p = 0.03). ( ): number of analyzed oocytes from at least three different experiments.

First, we measured cortical tension without any microinjection on oocytes at the VG stage and obtained an average of 3.574 ± 0.454 nN/μm. This value dropped drastically (*p* < 0.0001) with the oocyte maturation towards the MI matured *in vitro* (0.174 ± 0.020 nN/μm) or *in vivo* (0.142 ± 0.013 nN/μm) and MII stages matured *in vitro* (0.139 ± 0.008 nN/μm) or *in vivo* (0.091 ± 0.004 nN/μm). Comparisons of cortical tension between MI and MII oocytes *in vitro* and *in vivo* showed no significant differences (Figure 8A). Then we measured the cortical tension at the MII stage after *CTRL*, *ERM* or *EWI-2/EWI-F* siRNA injection at the VG stage and *in vitro* maturation during 24 h. Only *EWI-2/EWI-F* combined siRNA injection gave a significant decrease of the cortical tension from 0.109 ± 0.008 for the *CTRL* siRNA group to 0.069 ± 0.015 nN/μm (*p* = 0.02). Cortical tensions were comparable for the *ERM* and *CTRL* siRNA groups (*p* = 0.8, Figure 8B). When oocytes were matured *in vitro* during 48 h instead of 24 h the cortical tensions remained non-significantly different between the *CTRL* siRNA group (0.141 ± 0.006 nN/μm) and *ERM* siRNA group (0.116 ± 0.005 nN/μm; *p* = 0.9) but became very significant after injection of *EWI-2/EWI-F* siRNA (0.093 ± 0.005 nN/μm; *p* < 0.0001) or *Mix* (*EWI-2/EWI-F*–*ERM*) siRNA (0.078 ± 0.003, *p* < 0.0001). Moreover, there was no significant difference between the *EWI-2/EWI-F* and *Mix* siRNA groups (0.093 ± 0.005 versus 0.078 ± 0.003; *p* = 0.8) but the difference between *ERM* and *Mix* siRNA appeared significant (*p* = 0.03) (Figure 8C).

## Discussion

The aim of our study was to assess the role of the oocyte EWI-2, EWI-F and ERM protein family as potential partners of CD9 in gamete adhesion/fusion processes.

Our data showed that fertilization was not affected by the inhibition of one of these proteins, the fertilization index being not decreased after inhibition of either EWI-2, EWI-F, Ezrin, Radixin or Moesin, separately. Conversely, injection of *ERM* siRNA or combined *EWI-2/EWI-F* siRNA inhibited fertilization, decreasing the FI by 59 and 43% respectively. The lack of single siRNA effect did not appear to be due to a lack of siRNA efficiency since the injection of *EWI-F* or *EWI-2* siRNA induced significant decreases in protein expression (55 and 68% respectively), as well as *Radixin* siRNA alone caused a decrease in Radixin protein expression (56%) similar to that caused by the combined injection of *ERM* siRNA (62%). Thus, the lack of effect on fertilization could be explained by a redundancy mechanism, like already highlighted in the context of knockout gene experiments. Indeed, EWI-2 and EWI-F are both immunoglobulins known to be associated to tetraspanins in somatic cells as well as in oocytes (Charrin et al., 2001; Stipp et al., 2001; Charrin et al., 2003; Runge et al., 2007; Glazar and Evans, 2009; He et al., 2009). *EWI-2*-deficient mice have been demonstrated to be fertile. Their eggs retain the normal level and localization of CD9, resulting in normal microvilli formation (Inoue et al., 2012). While CD9 regulates the expression and localization of EWI-2, these results and ours indicate that EWI-2 and EWI-F do not regulate the expression and localization of CD9. These results also suggest a possible compensation by EWI-F. In addition, unlike the anti-CD9 antibody, an anti-EWI-2 antibody has moderate inhibitory effects on sperm-egg binding and fusion (Glazar and Evans, 2009). Similarly, in our previous experiments, a monoclonal antibody directed against the EWI-F (CD9P-1) molecule failed to inhibit fusion (Rubinstein et al., 2006). Otherwise, EWI-2 and EWI-F have been shown to associate directly to ERM in somatic cells (Sala-Valdes et al., 2006). ERM are thought to constitute a bridge between the actin cytoskeleton and the plasma membrane. *Ezrin*-deficient mice, that die by 3 weeks of age (explaining that their fertility cannot be explored), showed abnormalities in their intestinal epithelium morphogenesis with microvilli disorganization. It should be noted that Ezrin is the only ERM protein detected in the developing intestinal epithelium, and this is likely why no redundancy mechanism may occur in this case (Saotome et al., 2004). *Radixin*-deficient mice are viable and fertile (Kikuchi et al., 2002). *Moesin*-deficient mice are normal and fertile without any Ezrin or Radixin hyper-regulation (Doi et al., 1999). However, the mutations of the *Dmoesin* gene in *drosophila* have dramatic consequences on embryo development. Dmoesin is required during oogenesis for anchoring microfilaments to the oocyte cortex (Polesello et al., 2002), since in mutant oocytes, the subcortical actin network is detached from the cell membrane (Jankovics et al., 2002). This is consistent with the fact that no redundancy is possible in this non-mammalian species that lacks the *ezrin* and *radixin* genes. Indeed, ERM are encoded by three highly homologous genes that have ∼85% similarity at the amino acid level (For review (Louvet-Vallee, 2000; Fehon et al., 2010)).

Given that ERM proteins promote the CD9-cytoskeleton interaction (Sala-Valdes et al., 2006), we can hypothesize that ERM are also critical in gametes adhesion/fusion mechanism via this function. The fertilization inhibition obtained by siRNA injection was however less dramatic than the one we have reported in *Cd9*-knockout eggs (Jegou et al., 2011). This discrepancy could be due to the fact that although almost no pERM expression was detected by immunofluorescence after siRNA injection, it cannot be ruled out that few ERM proteins remained or that further ERM phosphorylation may take place. In this case, our results would underestimate their role. A significant decrease of protein expression necessitated 48 h after the injection of *EWI-2/EWI-F* siRNA, while 24 h post injection of *ERM* siRNA were sufficient to detect an important decrease suggesting that the EWI-2 and EWI-F proteins would be more stable than ERM proteins.

It has been shown that overexpression of ERMBMPs (ERM-binding membrane proteins) in collaboration with activated ERM proteins in several cell lines, induced formation and elongation of microvilli (Yonemura et al., 1999). A mixture of *ezrin*/*radixin*/*moesin* antisense oligonucleotides could induce the destruction of both cell-cell and cell-substrate adhesion, as well as the disappearance of microvilli (Takeuchi et al., 1994).

In addition, it has been shown that the mechanical properties of the cell cortex can predict the developmental potential of oocytes after fertilization in mice in humans (Yanez et al., 2016). Cortical tension is highly regulated during oocyte maturation and could also play a role during gamete adhesion/fusion.

In order to check if the functional finding, the fertilization decrease, resulted from microvilli morphological alteration and/or cortical tension deregulation, we measured the radius of curvature of microvilli on oocyte membranes and cortical tension after injection of *ERM* and/or *EWI-2/EWI-F* siRNA compared to *CTRL* siRNA. Indeed, the inhibition of the expression of these proteins also caused an increase in the radius of curvature of the oocyte microvilli and a decrease in cortical tension.

Several works have shown the link between the radius of curvature and fusion. A small radius of curvature at the tip of microvilli maximizes the efficiency of membrane fusion (Wilson and Snell, 1998; McMahon and Gallop, 2005). In several cases of cell fusion, a small radius of curvature of the membrane protrusions promotes fusion (Wilson and Snell, 1998). Similarly, the fusion efficiency is higher when vesicles have a smaller radius of curvature (Lentz et al., 1992). Otherwise, several studies showed the involvement of Ezrin in microvilli morphology (Saotome et al., 2004; Bonilha et al., 2006). Moreover, in accordance with our results, in thymoma cells cultured in presence of antisense oligonucleotides complementary to *ERM* sequences, microvilli disappear, whereas when each one of the *ERM* sequences was inhibited separately, microvilli remain (Takeuchi et al., 1994). Finally, the involvement of oocyte microvilli in gamete interaction is strongly suggested by the fact that this interaction has been reported to be restricted to the microvilli-rich region (Yanagimachi, 1978; Runge et al., 2007). In addition, consistently with our results, the radius of curvature of microvillar tips on wild-type oocytes was found to be half that of the *Cd9*-null oocytes (Runge et al., 2007). But contrary to our results, *Cd9*-knockout oocytes were also shown to have denser arrays of microvilli than wild-type oocytes. Surprisingly, we did not observe any difference in terms of density of microvilli after injection of *ERM* or *EWI-2/EWI-F* siRNA whereas this decreased after the combined injection of the two (*Mix* siRNA). This result seems to indicate that ERM and EWI-2/EWI-F would be involved in the formation of microvilli while CD9 would negatively regulate this one, probably by acting indirectly on the organization of other proteins. The absence of this negative regulation in the context of *Cd9*-KO could explain microvilli density increase.

If a correlation between the increase in the radius of curvature of the microvilli and the decrease in the fertilizing ability of the oocytes seems to be rational and very likely (Runge et al., 2007), the link between the decrease in cortical tension and that in the fertilization index is more questionable. Cortical tension measures the mechanical characteristics of both the membrane and the subcortical cytoskeleton. But the major contribution of cortical tension comes from the cytoskeleton. Therefore, an inhibition of the links between the two may result in a slight drop in cortical tension. Indeed, the difference we obtained in terms of cortical tension between the oocytes microinjected by *CTRL* siRNAs and those microinjected by *EWI-2/EWI-F* and *ERM* siRNAs were too small to explain the important difference in fertilization index between these two groups. Especially since even more important differences of cortical tension, such as those observed between GV and metaphase I or metaphase II oocytes do not completely prevent these oocytes, whatever their stage of maturation, from fusing with sperm (Kryzak et al., 2013). However, it is possible that optimal adhesion/fusion during fertilization requires a narrow range of cortical tension, but additional investigations are necessary to prove it.

Since we cannot totally exclude the existence of other molecules able to overcome the lack of EWI-2/EWI-F or ERM proteins, the confirmation of redundancy mechanism on one hand between EWI-2 and EWI-F and on the other hand between Ezrin, Radixin and Moesin remains to be provided by experiments using *EWI-2* and *EWI-F* double-knockout mice and *ERM* conditional oocyte specific triple-knockout mice respectively.

In conclusion, we highlighted here the role of oocyte EWI-2, EWI-F and ERM in fertilization, specifically in the step of gamete adhesion/fusion. The ERM and EWI-2/EWI-F proteins seem involved in gamete interaction through their role in the formation and maintenance of functional oocyte microvilli. These results confirm the role of microvilli in gamete interaction and support the hypothesis of the existence of a link between CD9 and the cytoskeleton through EWI-2/EWI-F and ERM proteins.

## Data Availability

The original contributions presented in the study are included in the article, further inquiries can be directed to the corresponding author.
